# Artists and eating disorders: what do we know? A systematic review of the evidence and gaps

**DOI:** 10.1007/s40519-026-01863-3

**Published:** 2026-05-05

**Authors:** Emma Bui, Musa Jemal Negash, Georgia Burnham, Jia Xian Liew, Nhi Tran Phuong, Isabel Krug

**Affiliations:** Melbourne School of Psychological Sciences, University of Melbourne, Redmond Barry Building, Room 70, Parkville, VIC 3010 Australia

**Keywords:** Eating disorders, Artists, prevalence, Risk, Body dissatisfaction, General psychopathology, Systematic review, Meta-analysis

## Abstract

**Objectives:**

Although artists are often considered at higher risk for eating disorders (EDs), evidence remains limited. We therefore conducted a systematic review of ED occurrence, risk, body image concerns, and general psychopathology in artistic populations.

**Method:**

Following PRISMA guidelines, PsycINFO, Medline, and Web of Science were searched for English-language studies published in any year. Fifteen studies met inclusion criteria and were narratively synthesised.

**Results:**

The review encompassed a broad range of artistic groups (e.g., musicians, actors, circus and drag performers, and theatre artists). Four studies reported lifetime ED diagnoses (2.9% to 22.5%; mean ≈ 12.1%), while three studies assessed current ED diagnoses (1.4% to 18.7%; mean ≈ 7.5%). Six studies reported ED risk levels, which varied substantially (8% to 70.7%; mean ≈ 27.8%), reflecting considerable heterogeneity in samples and methodologies. Importantly, these estimates were largely derived from self-report or non-standardised assessments rather than validated structured clinical interviews, limiting their interpretability and comparability. Despite these limitations, most studies indicated elevated ED-related indicators and body dissatisfaction in artistic populations relative to general population benchmarks, although such comparisons should be interpreted with caution. Across studies, body image concerns were consistently associated with ED symptoms, and anxiety and depression were positively related to eating pathology.

**Discussion:**

Artists may represent a population at elevated risk for eating- and body-related psychopathology. However, methodological limitations and heterogeneity highlight the need for more rigorous, diverse, and longitudinal research to clarify mechanisms of vulnerability and inform targeted prevention and early intervention.

**Level of evidence:**

Level I, systematic review and meta-analysis.

**Supplementary Information:**

The online version contains supplementary material available at 10.1007/s40519-026-01863-3.

## Introduction

Eating disorders (EDs) arise from a complex interplay of sociocultural, familial, and individual factors [1, 2]. Cultural ideals of thinness and familial reinforcement of disordered eating exert strong influences, while temperament, low self-esteem, and maladaptive cognitions contribute to their onset and maintenance [1, 3]. Certain groups face heightened exposure to these risks—among them, artists working across visual, musical, and performing arts [4, 5]. Evidence suggests that artists display elevated emotional and cognitive vulnerabilities linked to ED risk. They report higher levels of depression, anxiety, and stress—particularly among visual artists [6]—and those with lower self-appraisals of artistic ability, perfectionism, and self-criticism show reduced self-esteem [7–9]. Low self-esteem, in turn, has been shown to predict ED symptoms and to amplify the impact of perfectionism on disordered eating [10, 11]. Collectively, these findings highlight the accumulation of affective and cognitive vulnerabilities that may render artists particularly susceptible to eating pathology.

Comorbidity between mood disorders and EDs further underscores this risk in the artist population [12]. Taylor [13] found higher rates of major depressive and bipolar disorders among artists across writing, visual arts, performance, and music compared to non-artists. Negative mood and ED symptomatology frequently co-occur, with each potentially functioning as a precursor for the other [14, 15]. The higher prevalence of mood problems among artists may therefore signify both an increased risk of ED onset and the possibility that eating pathology contributes to elevated mood disorder rates in this group. This intersection highlights the need to examine the prevalence and clinical features of EDs in artists to inform prevention and intervention approaches sensitive to their unique vulnerabilities [4, 5].

Despite this elevated risk profile, most research on EDs in artistic populations has focused narrowly on dancers. Arcelus et al. [16] for instance demonstrated significantly higher rates of EDs among dancers compared to non-dancers, as well as distinct patterns of eating pathology and general psychopathology. These findings were attributed to dancers’ heightened body-focused demands, mirroring similar risks observed in athletes [17]. Yet, whether similarly elevated levels of ED symptoms and diagnoses extend to artists outside of dance—where body image is less central to performance—remains unclear. Investigating ED occurrence in these groups is crucial to determine whether individual-level vulnerabilities alone are sufficient to elevate risk, or whether body-focused occupational demands are a primary driver.

Evidence beyond dance remains scarce. Kapsetaki and Easmon [4] systematically reviewed 10 studies of non-dance performing artists (e.g., musicians, actors) and noted inconsistent findings, compounded by small sample sizes and methodological variability. However, this review was conducted 9 years ago and may no longer reflect the current state of the evidence base. Importantly, the review did not include a systematic quality appraisal of the included studies, limiting the validity of their conclusions. The lack of rigorous synthesis and comparison across studies has hampered the field’s ability to meaningfully interpret ED prevalence and symptomatology trends in artists and to translate findings into clinical practice.

No structured review has yet comprehensively examined ED presentations, risk, and related psychopathology (e.g., anxiety, depression) in the broader artist population. This represents a critical gap, given evidence of elevated ED-related risk factors across creative domains beyond performance art. Addressing this gap is essential not only to characterise the scope and clinical features of EDs in these groups, but also to guide resource allocation, prevention strategies, and tailored treatment approaches for artists.

## The current review

In the present review, the term “artist” is used in a broad and inclusive sense, encompassing both professional artists and individuals engaged in artistic or performance-based training (e.g., circus artists, drag performers, and marching band students). This approach was adopted because the existing literature in this area is sparse and fragmented, with studies rarely focusing exclusively on narrowly defined professional artist populations. Importantly, these groups share several features that are theoretically relevant to EDs, including public performance, appearance-based evaluation, and performance-related pressures; nevertheless, this broad categorisation inevitably introduces heterogeneity, and the findings should be interpreted with this limitation in mind.

Given the limited and methodologically diverse nature of the existing literature, the present review is positioned as a state-of-the-literature overview aimed at synthesising current evidence and identifying key gaps, rather than providing precise estimates of ED risk or occurrence in artists.

Against this background, the present review aimed to (1) systematically synthesise the literature on EDs (including ED risk), body dissatisfaction, and associated psychopathology (e.g., mood and anxiety) across a wide range of artistic fields; and (2) conduct a quality appraisal of the included studies.

Rather than testing a directional hypothesis, this review takes an exploratory approach to characterising patterns of ED-related outcomes, body image concerns, and associated psychopathology in artistic populations, while critically evaluating the strength and limitations of the available evidence.

## Methods

### Protocol and registration

This systematic review was conducted in accordance with the Preferred Reporting Items for Systematic Reviews and Meta-Analyses (PRISMA) guidelines [18] and was prospectively registered with the International Prospective Register of Systematic Reviews (PROSPERO) in March 2025 (registration number: CRD420251010427).

### Literature search

A comprehensive search was performed across PsycINFO, Medline, and Web of Science on 16 March 2025 and updated on 24 April 2025. Search terms combined keywords relating to eating disorders (e.g., *eating disorder*, *anorexia nervosa*), body image (e.g., *body dissatisfaction*, *body image*), and the target artist population (e.g., *musician*, *artist*) using Boolean operators. No restrictions were placed on publication date. The full search strategy is provided in the Supplementary Material 1.

### Eligibility criteria

Studies were eligible if they (a) included a sample or sub-sample of professional or university-level artists or individuals engaged in artistic or performance-based training (e.g., visual arts, music, theatre, acting, performing arts, writing, or architecture); (b) reported on the occurrence of ED diagnoses, risk for EDs, or ED symptomatology (including body image dissatisfaction); and/or (c) used standardized measures of eating pathology, body image, or general psychopathology.

This inclusive approach, encompassing professional artists, students in artistic training, and performance-based groups, was adopted to capture the full scope of the available evidence given the limited and heterogeneous nature of the literature. We acknowledge that some included samples overlap conceptually with student or athletic populations; however, studies were retained if participants’ primary activity involved artistic or performance-based practice.

Studies were excluded if they: (a) exclusively examined dancers, athletes, or other groups not specified above; (b) sampled general populations without identifying an artist sub-sample; (c) focused solely on Avoidant/Restrictive Food Intake Disorder (ARFID), pica, rumination disorder, or orthorexia nervosa; (d) were opinion papers, book chapters, qualitative studies, systematic reviews, meta-analyses, or case studies; (e) investigated art-, dance-, or music-therapy interventions; or (f) were not published in English.

### Study screening

Search results were imported into Covidence systematic review software, and duplicates were removed both automatically and manually. Given the large volume of records, title and abstract double-screening was initially conducted by two independent reviewers E. B. and G. B., with an additional two reviewers N. T. and J. L added later. Approximately 50% of records were randomly double screened across the four reviewers, with the rest of the studies continued to be independently screened by the four reviewers.

In the title and abstract screening phase, the agreement between reviewers G. B. and J. L was minimal (*κ* = 0.36), between E. B. and G. B was weak (*κ* = 0.57), between E. B. and J. L was minimal (*κ* = 0.36), and no agreement between J. L and N. T (*κ* = − 0.01) based on McHugh’s [19] interpretation.

**Fig. 1:**
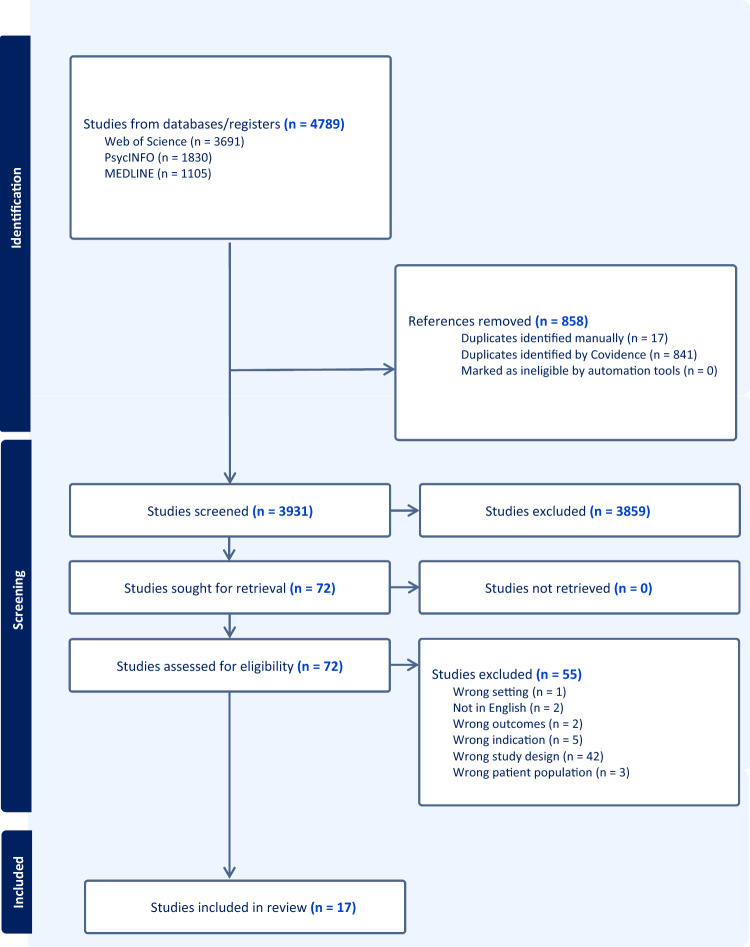
PRISMA Flow diagram of screening process

Studies that met inclusion criteria progressed to full-text review, which was conducted independently by the reviewers, with 50% of texts double-screened by all four reviewers. Any discrepancies were resolved through discussion. In the full-text review phase, the agreements between reviewers E. B. and G. B, J. L and N. T, and E. B. and N. T were perfect (*κ* = 1.00), while there was no agreement between E. B. and J. L (*κ* = 0.00; [19]). A PRISMA flow diagram illustrating the screening process is presented in Fig. 1.

No additional procedures were implemented at this stage beyond the initial calibration exercise conducted at the outset. In response to this, a more thorough calibration exercise was conducted prior to full-text screening, with further clarification of inclusion and exclusion criteria to enhance consistency and reliability in study selection at this stage.

The low inter-rater reliability observed across both screening stages was primarily attributable to ambiguous reporting within the included studies. It was often unclear whether data were collected using qualitative or quantitative methods, and in some cases, studies may have contained relevant data that were not explicitly reported in the title or abstract because these were not the primary focus of the research. Discrepancies at both screening stages were addressed through detailed discussions among all four reviewers, with final inclusion or exclusion decisions determined by majority consensus (agreement of at least three of the four reviewers).

### Data extraction

Two reviewers independently extracted data into a standardized Excel spreadsheet. Extracted data included: (a) study-level details (title, corresponding author, country); and (b) participant characteristics (sample size, mean age, gender, ethnicity, artistic field, ED diagnosis or ED risk occurrence, and control group information where applicable). During this stage, two additional studies [20, 21] were excluded due to ineligible populations. Where relevant data were missing, study authors were contacted up to three times for clarification or additional information.

### Quality appraisal

Methodological quality was independently assessed by two reviewers using the Joanna Briggs Institute (JBI) critical appraisal tool for analytical cross-sectional studies [22]. Each study was evaluated against eight criteria, scored as 2 (“yes”), 1 (“unclear”), 0 (“no”), or not applicable. Based on cumulative scores, studies were classified as high, good, or low quality. Appraisal of all the studies was double screened by reviewers G. B. and N. T. Disagreements were resolved through discussion alongside reviewer E. B., achieving an overall agreement rate of 86.7% across the eight criteria and a moderate inter-rater agreement (*κ* = 0.77; [19]).

## Results

### Study selection

The database searches yielded 4789 records. After the removal of duplicates, 3931 unique studies were screened by title and abstract. Of these, 72 articles underwent full-text review, resulting in 17 studies meeting inclusion criteria. Two additional studies [20, 21] were excluded at the data extraction stage due to ineligible populations being female university students and being female models and athletes respectively, leaving 15 studies in the final review.

Of the 15 included studies, eight were eligible for inclusion in the meta-analysis examining the prevalence of ED risk. However, given the substantial heterogeneity in study populations, assessment tools, and methodological approaches, the pooled estimates should be interpreted with caution. Detailed meta-analytic findings are therefore presented as preliminary in Supplementary Material 2.

### Study characteristics

Table 1 provides an overview of the included studies, including country of origin, ED diagnostic approach, comparison groups, sample size, participant demographics (age, gender, ethnicity), and outcome measures.

**Table 1:** Characteristic overview of included studies in the systematic review

Author/s and year	Country	Diagnosis method	Comparison group/s	Sample size	[Age range]; Mean age (SD)	Gender	Ethnicity/race	Outcome Measures
Carretta et al., [33]	United States	NA	Drag queens	192	[18–63]; 29.5 (11.1)	Male = 159; genderqueer/gender non-conforming: 19; trans: 2; different identity: 12	White: 72%, Latino/a: 11%, African American/Black: 4%, Asian American/Pacific Islander: 4%, Native American/Alaskan Native: 1%, Biracial/Multiracial: 7%, Other: 1%	EAT-26; UDACS
Colyer, [34]	United States	NA	Opera singers; CP	124	[18–69]; 32.8 (10.6)	Female = 124; male = 34; non-binary = 11	White: 79.0%; Asian: 5.6%; Latinx: 40%; Black/African American: 4.8%; Middle Eastern: 2.4%; Southeast Asian: 1.6%; Other: 2.4%	SHAI; EAT-26; BESAA
DiPasquale, [35]*	United States	Self-report measure (QEDD + BULIT-R)	Musicians; CP	Whole sample = 669; musicians = 219; CP = 450	[18–30]; NR	Female = 93; male = 126	NR	QEDD; BULIT-R
Emerson et al., [23]	United States	NA	Collegiate marching band	1207	[18–35]; 19.6 (1.3)	Female = 48; male = 26	Asian: 2.7%; Black or African American: 4.1%; Hispanic or Latino: 2.7%; White: 78.4%' Multi-racial: 8.1; Other: 4.1%	NA
Greenspan & Stuckey, [31]	United States	Self-report	Circus artists	201	[13–69]; 31.4 (8.9)	Female = 172; male = 48	NR	NA
Joseph et al., [28]*	United States	Self-report	Drama students – AN; CP—AN	Whole sample = 86; drama students = 40; CP = 46	[NR]; Drama = 20.9 (NR); CP = 27.7 (NR)	NR	NR	EAT
Kapsetaki & Easmon, [36]*	United Kingdom	Self-report; self-report measure (EDE-Q)	Musicians – AN; musicians – BN; musicians – BED	306	[18–75]; 31.4 (12.5)	AN: Female = 15, male = 6; BN: Female = 16, male = 4; BED: Female = 19, male = 9	NR	DASS-21; EDE-Q
Kelly et al., [24]	United States	Self-report	Collegiate marching band	1207	[18–35]; 19.6 (1.3)	Female = 684; male = 511; male-to-female = 1; female-to-male = 6	White: 82.9%; Black or African American: 3.6%; Asian: 3.0%; Hispanic or Latino: 2.7%; Multiracial: 6.2%; Other: 1.7%	NA
Rens et al., [32]*	Australia	NA	Circus artists	500	[18–69]; 31.1 (8.2)	Female = 415; male = 62; transgender/gender diverse = 23	NR	EAT-26
Szabo et al., [29]*	Australia	Self-report measure (EDDS)	Actors/performing artists – AN; Actors/performing artists – BN; Actors/performing artists – BED	573	[15.5–83]; 41.8 (14.3)	Female = 325; male = 248	NR	EDDS
Uriegas et al., [25]*	United States	NA	University marching band	150	[18–23]; 19.9 (1.1)	Female = 84; male = 66	White: 80.7; Hispanic or Latino: 2.7%; Black or African American: 5.3%; American Indian or Alaska Native: 0.7; Other: 8.7%	EDI-3
Vitzthum et al., [26]*	Germany	NA	Musical theatre students	37	[17–26]; 21.3 (2.1)	Female = 17; male = 20	NR	EDE-Q
Walton et al., [30]	Australia	NA	Musicians; actors	Whole sample = 143; musicians = 111; actors = 32	[> 18]; 32.7 (13.4)	Female = 131; male = 62; non-binary/other = 18	Aboriginal or Torres Strait Islander: 8%	GAD-7; BAS-2
Werner et al., [27]	United States	NA	Drama students; musical theatre students	Whole sample = 54; drama students = 18; musical theatre students = 36	[17–24]; 20.3 (3.5)	Female = 48; male = 23	White: 97%	BIS
Woropay-Hordziejewicz et al., [37]*	Poland	NA	Music students	255	[NR]; 23.1 (3.5)	Female = 184; male = 71	NR	HADS; EAT-26

*Studies included in the meta-analysis

AN = Anorexia Nervosa; BN = Bulimia Nervosa; BED = Binge-Eating Disorder; CP = Control Participant; BAS-2 = Body Acceptance Scale 2; EDE-Q = Eating Disorder Examination-Questionnaire; SHAI = Short Health Anxiety Inventory; GAD-7 = Generalised Anxiety Disorder 7; EDI-3 = Eating Disorder Inventory-3; EAT-26 = Eating Attitudes Test 26; EAT = Eating Attitudes Test; QEDD = Questionnaire for Eating Disorder Diagnosis; BULIT-R = Bulimia Test Revised; DASS-21 = Depression Anxiety Stress Scale 21; HADS = Hospital Anxiety and Depression Scale; EDDA = Eating Disorder Diagnostic Scale; UDACS = Upward and Downward Appearance Comparison Scale – upward appearance comparison scale; BESAA = Body-Esteem Scale for Adolescents and Adults; BIS = Body Image Scale; NR = Not Reported; NA = Not Applicable

The included studies comprised a total of 4047 artists, with sample sizes ranging from 37 to 1207 participants. The mean participant age was 26.8 years (range: 13–83). The artistic populations investigated included musicians (n = 5, including singers), university/collegiate marching band members (n = 3; [23–25]), musical theatre performers (n = 2; [26, 27]), drama performers (n = 2; [27, 28]), actors (n = 2; [29, 30]), circus artists (n = 2; [31, 32]), and drag performers (n = 1; [33]).

Ten studies [25, 26, 28, 29, 32–37] assessed eating pathology, most commonly via the Eating Attitudes Test (EAT-26 or EAT; [38, 39]; n = 5; [28, 32–34, 37]), followed by the Eating Disorder Examination Questionnaire (EDE-Q; [40]; n = 2; [26, 36]), the Questionnaire for Eating Disorder Diagnoses (QEDD; [41]; n = 1; [35]) combined with the Bulimia Test–Revised (BULIT-R; [42]; n = 1; [35]), the Eating Disorder Diagnostic Scale (EDDS; [43]; n = 1; [29]), and the Eating Disorder Inventory–3 (EDI-3; [44]; n = 1; [25]).

In terms of body image concerns, four studies [25, 30, 33, 34] assessed body dissatisfaction, each employing a different instrument: the Body Image Subscale (BIS; [45]) of the Offer Self-Image Questionnaire [27, 45], the Body Appreciation Scale–2 (BAS-2; [30, 46]), the Body Esteem Scale for Adolescents and Adults (BESAA; [34, 47]), and the Upward Appearance Comparison subscale of the Upward and Downward Appearance Comparison Scale (UDACS; [33, 48]).

General psychopathology (including mainly depression and anxiety) was assessed in four studies [30, 34, 36, 37], each using a distinct measure: the Hospital Anxiety and Depression Scale (HADS; [37, 49]), the Generalized Anxiety Disorder Scale (GAD-7; [30, 50]), the Depression Anxiety Stress Scales (DASS-21; [36, 51]), and the Short Health Anxiety Inventory (SHAI; [34, 52]).

Six studies [24, 28, 29, 31, 35, 36] reported ED diagnoses, all derived from self-report measures, and six studies examined ED risk [23, 25, 26, 28, 32, 37]. Two studies [29, 36] examined subtype-specific diagnoses of anorexia nervosa (AN), bulimia nervosa (BN), and binge-eating disorder (BED), and one study [28] examined risk of AN specifically.

### Synthesis of findings

Table 2 summarizes the primary outcomes of all the included studies, including estimates of occurrence and trends in eating pathology and body dissatisfaction. Four studies [24, 28, 31, 36] assessed lifetime ED diagnoses, reporting proportions between 2.9% and 22.5% (mean ≈12.1%). Three studies [29, 35, 36] examined point estimates, ranging from 1.4% to 18.7% (mean ≈ 7.5%). Finally, across six studies [23, 25, 26, 28, 32, 37] investigating ED risk, between 8% and 70.7% of participants were classified as at risk for an ED, with a mean of 27.8%.

**Table 2:** Main findings and outcomes of included studies

Study	Main findings in the artist populations	Prevalence n (%)
Carretta et al., [33]	• Hyper-feminine drag → body image concerns, disordered eating, upward appearance comparison	• NA
Colyer, [34]	• Body esteem → healthy anxiety • ↓Body esteem, ↑health anxiety in singers vs. non-singers	• NA
DiPasquale, [35]	• Prevalence rates in music = non-music students	• ED point prevalence: Artists = 5 (2.3); CP = 29 (6.4)
Emerson et al., [23]	• Nutrition behaviours → exercise-associated hyponatremia	• Perceived ED rate: 16 (21.6)
Greenspan & Stuckey, [31]	• ED → higher injury risk	• Lifetime ED rate: 36 (18)
Joseph et al., [28]	• EAT mean = controls • AN risk ↑ in drama students	• ED risk rate: Artists = 8 (20), CP = 4 (8) • Lifetime prevalence rate: Artists = 2 (5)
Kapsetaki & Easmon, [36]	• ↑ED prevalence/EDE-Q • Weak links to perfectionism, depression, anxiety, and stress	• Lifetime prevalence rate: Overall = 69 (22.5), AN = 21 (8.1), BN = 20 (7.7), BED = 28 (10.8) • ED point prevalence: 57 (18.7)
Kelly et al., [24]	• Exertional heat illness → nutrition behaviours, ED diagnosis/perception	• Lifetime prevalence rate: 35 (2.9)
Rens et al., [32]	• ↑ risk of ED; ED exercise addiction • Psychological resilience ↓ likelihood of ED	• ED risk rate: 178 (35.6)
Szabo et al., [29]	• AN: 0 in males, ↑ in females vs general population • BN: ↑ in both sexes’ vs general population • BED = general population	• ED point prevalence rate: Overall = 95 (16.6), AN = 8 (1.4), BN = 64 (11.2), BED = 23 (4)
Uriegas et al., [25]	• ED risk ≥ general student population	• ED risk rate: 106 (70.7)
Vitzthum et al., [26]	• ↓ Nutrition related knowledge • ↑ED risk	• ED risk rate: 3 (8)
Walton et al., [30]	• Self-compassion →↑ mental health; stronger association with mental health outcomes than body appreciation	• NA
Werner et al., [27]	• Healthy body image; ↓ purging behaviours;	• NA
Woropay-Hordziejewicz et al., [37]	• Prevalence of ED in those with study addiction > general population • Study addiction and ED highly comorbid with depression and anxiety	• ED risk rate: 27 (10.6)

AN = Anorexia Nervosa; BN = Bulimia Nervosa; BED = Binge-Eating Disorder; CP = Control Participant; ED = Eating Disorders; NA = Not Applicable

However, it is important to note that these estimates were derived from self-report or self-administered screening instruments and were not verified using validated structured clinical interviews such as the Structured Clinical Interview for Diagnostic and Statistical Manual of Mental Disorders (SCID; [53]) or Eating Disorder Examination ([40]), which limits their interpretability and comparability across studies.

With respect to ED symptomatology, although the EAT-26 [38] was employed in nearly half of the studies assessing eating pathology, only two studies [33, 34] reported sufficient data to calculate mean scores. The reported mean scores in these studies ranged from 2.4 to 9, with standard deviations ranging from 0.7 to 11, which is considered low as they are below the cut-off score of 20 in the EAT-26 for having an ED risk [38].

Of the 11 studies that provided comparative or rate-based data (excluding correlational analyses only; [25–29, 31, 32, 34–37]), eight studies [25, 26, 28, 29, 32, 34, 36, 37] reported elevated rates of ED risk and/or body dissatisfaction among artists compared to the general population estimates, while three studies [27, 31, 35] reported no significant differences between artists and non-artists.

Four studies specifically examined body image concerns [27, 30, 33, 34], which were generally found to correlate with ED symptomatology. Regarding general psychopathology, only one study [34] compared artists and controls, reporting significantly higher health anxiety among artists. Three additional studies [30, 36, 37] assessed overall levels of anxiety and depression, both of which were positively associated with ED and body dissatisfaction symptoms.

### Quality appraisal

Quality appraisal cumulative scores of the studies ranged from 6 to 16, with a mean of 9.5. Based on the range of ratings studies scored on the JBI critical appraisal checklist [22], studies were classified as low (6–9), good (10–13), or high quality (14–16). Table 3 shows individual study quality ratings for each criterion and cumulative scores. Nine studies were rated as low quality [23, 24, 26–28, 30, 31, 33, 37], four as good quality [25, 29, 32, 36], and two [34, 35] as high quality. While the most common reason for low quality was because confounding factors related criteria was not applicable for most studies (12 out of 15 studies; with the exception of [34–36]), the main shortcoming was the lack of valid and reliable measure of outcome (5 out of 15; [23, 24, 27, 29, 31]).

**Table 3:** Quality appraisal consensus and overall scores of included studies

Author/s and year	C 1	C 2	C 3	C4	C5	C 6	C7	C8	Overall score
Carretta et al., [33]	1	2	2	0	NA	NA	2	2	**9**
Colyer, [34]	2	2	2	2	2	0	2	2	**14**
DiPasquale, [35]	2	2	2	2	2	2	2	2	**16**
Emerson et al., [23]	2	2	2	2	NA	NA	0	1	**7**
Greenspan & Stuckey, [31]	2	2	0	1	NA	NA	0	2	**6**
Joseph et al., [28]	0	0	2	2	0	0	2	2	**8**
Kapsetaki & Easmon, [36]	2	2	2	0	NA	NA	2	2	**10**
Kelly et al., [24]	2	2	0	2	NA	NA	0	2	**8**
Rens et al., [32]	0	2	2	2	NA	NA	2	2	**10**
Szabo et al., [29]	1	2	2	2	NA	NA	1	2	**10**
Uriegas et al., [25]	2	2	2	2	NA	NA	2	2	**12**
Vitzthum et al., [26]	0	2	2	2	NA	NA	2	0	**8**
Walton et al., [30]	0	2	2	1	NA	NA	2	2	**9**
Werner et al., [27]	0	2	0	2	NA	NA	0	2	**6**
Woropay-Hordziejewicz et al., [37]	0	2	2	1	NA	NA	2	2	**9**

NA = Not Applicable C1: Clearly defined inclusion criteria; C2: Detailed study subjects and setting; C3: Valid and reliable measure of exposure; C4: Objective, standard criteria used for measurement of condition; C5: Confounding factors identified; C6: Strategies to deal with confounding factors; C7: Valid and reliable measure of outcomes; C8: Appropriate statistical analysis

## Discussion

This review synthesised a small and methodologically heterogeneous literature on ED diagnoses, ED risk, body image concerns, and anxiety and depression across diverse artistic populations, including musicians, actors, circus and drag performers, and theatre artists. Lifetime ED diagnoses were assessed in four studies, with estimates ranging from 2.9% to 22.5% (mean ≈ 12.1%), while current diagnoses were examined in three studies, ranging from 1.4% to 18.7% (mean ≈ 7.5%). Estimates of ED risk varied widely across six studies (8–70.7%; mean ≈ 27.8%).

Although most studies reported higher ED risk, rates, and/or body dissatisfaction in artists than in general population samples, these findings are highly inconsistent and must be interpreted cautiously. Nine of the fifteen studies were rated as low quality, several relied on small samples, unclear cut-offs, or overlapping datasets, and most outcomes were based on self-report screening instruments rather than structured diagnostic interviews. Accordingly, the evidence is better interpreted as indicating possible elevated vulnerability rather than providing reliable estimates of clinical occurrence.

Importantly, synthesising findings across diverse artistic groups assumes a coherent population, yet it remains unclear whether these groups share meaningful occupational or sociocultural risk factors relevant to EDs. The category “artist” spans disciplines with markedly different performance demands, appearance pressures, and cultural norms. As such, treating artists as a unified epidemiological group may obscure important subgroup differences and limit the interpretability of aggregated findings. This highlights the need for more theoretically grounded classification of artistic populations in future research.

Despite the widespread use of the EAT-26 as a screening tool, few studies (e.g., [33, 34]) reported continuous outcomes, limiting conclusions regarding ED symptom severity. Furthermore, very few studies assessed body image, anxiety or depression, and most studies lacked control groups, constraining the ability to draw meaningful comparisons with non-artist populations and underscoring the need for more comprehensive and methodologically robust research in this area.

Lifetime rates of ED diagnoses in the included studies ranged from 2.9% to 22.5% (mean ≈12.1%), which is higher than the general population estimates of 0.19% [54]. However, it is important to note that none of these studies verified ED diagnoses using validated structured clinical interviews (e.g. SCID or EDE) instead, diagnoses were based on self-report or non-standardised assessment methods, which limits the interpretability and comparability of these estimates. In contrast, current estimate rates (mean ≈7.5%) were comparable to or lower than the community sample rate of 17.0% reported in the meta-analysis by [55].These findings suggest that although active ED diagnoses at a given time point may not exceed population norms, artists appear to carry a higher cumulative lifetime burden of EDs.

While the average ED risk estimate appeared to be similar to rates reported among university students [56], the breadth of the range suggests that certain artistic subgroups may be disproportionately affected. For instance, studies involving musicians [34, 36, 37] and musical theatre performers [26] reported higher proportions of participants exceeding ED risk thresholds compared to actors [28, 29] or circus performers [31, 32]. This variability may reflect differences in occupational demands, cultural pressures, or training environments across art forms, and suggests that artistic populations do not constitute a single, unified risk group but rather a collection of distinct subpopulations with different pathways to risk.

The patterns of higher ED risk and estimates observed in artists compared with non-artist populations are broadly consistent with evidence suggesting that artists may be more likely to show enduring individual-level vulnerabilities such as negative affectivity, perfectionism, and low self-esteem [6–9]. These characteristics may be linked to heightened sensitivity to evaluation, rigid performance standards, and difficulties with emotion regulation, which could increase susceptibility to disordered eating as a maladaptive coping strategy. Over time, such vulnerabilities may contribute not only to the emergence of eating pathology but also to its persistence or recurrence, potentially increasing the likelihood of repeated episodes or more chronic illness trajectories, although direct causal pathways cannot be established from the current evidence.

Accordingly, Stuckey et al. [5] reported that among circus artists, disordered eating—although associated with poorer mental health and higher perfectionism—may in some contexts be perceived as reflecting discipline or commitment within a performance culture that values leanness. Such interpretations could inadvertently reinforce these behaviours, which in turn may be associated with reduced energy availability and potential negative effects on performance. Over time, this pattern may also interact with underlying mental health difficulties and perfectionistic tendencies. Stuckey et al. [5] further suggested that factors such as professional status, age, gender, artistic discipline, and coping with stress and injury may also play a role in shaping these relationships.

Finally, the assessment of body dissatisfaction and anxiety and depression was limited and highly heterogeneous across studies. Out of the 15 studies, only four studies [27, 30, 33, 34] assessed body dissatisfaction, and they all used different measures (Upward Comparison subscale; BESAA; BAS2 and BIS). Similarly, only four studies [30, 34, 36, 37] accounted for general psychopathology and used different measures to assess depression and anxiety symptoms (SHAI, DASS-21, GAD-7, HADS). This extreme heterogeneity of assessment measures thus limited the potential for meta-analysis of these factors and their relationship with EDs in artists.

Overall, this review suggests that individual risk factors may play an important role in the elevated levels of ED–related symptoms and risk observed in some artist samples. Given that artists are generally thought to be less directly exposed to appearance-focused body ideals than groups such as dancers or athletes, the observation of comparably elevated screening-based risk in these populations [16, 17] raises the possibility that shared characteristics such as negative affect and perfectionism could contribute to vulnerability in eating habits and body-related self-perception. However, these interpretations remain tentative and cannot establish causality, particularly considering the methodological limitations of the current evidence base.

## Strength and limitations

Several limitations warrant careful consideration. First, the included populations ranged from professional artists to students and performance-based groups, some of whom may more closely resemble athletic or general student samples than established professional artists. While this reflects the current state of the literature, it increases heterogeneity and limits the precision with which conclusions can be drawn about “artists” as a unified group. Importantly, the validity of “artists” as an epidemiological category remains uncertain, as grouping diverse disciplines may obscure meaningful differences in occupational exposure, sociocultural context, and performance-related pressures that are likely relevant to eating disorder risk. As such, the assumption of a shared risk profile across artistic populations should be treated with caution.

In addition, no included studies investigated visual artists, writers, or architects, limiting generalisability to the broader creative community. Future research should extend to underrepresented artistic domains and adopt more clearly defined and stratified groupings, as current evidence remains largely restricted to performing artists (e.g., musicians, actors, circus performers), thereby constraining a more comprehensive understanding of eating disorder risk across the arts.

Second, another key limitation of the current literature is the overall methodological quality of the evidence base. Nearly all included studies relied on self-report screening instruments and cut-off scores rather than validated structured diagnostic interviews (e.g., the SCID or EDE). As a result, most estimates reflect screening-based risk or probable cases rather than confirmed clinical ED diagnoses and therefore cannot be interpreted as true prevalence of EDs. Such reliance on screening measures increases the risk of misclassification, reduces diagnostic validity, and undermines the precision of prevalence estimates [57]. In addition, several studies were rated as low quality, with small sample sizes, missing data (e.g., [28]), overlapping samples (e.g., Emerson et al. [23] reporting a subset of Kelly et al. [24]), or unclear cut-off points [25, 29, 35]. Collectively, these methodological weaknesses reduce confidence in the reliability of the evidence base. Future research should prioritise larger, higher-quality studies that employ validated diagnostic tools and transparent reporting.

Third, although this review employed systematic methods, the database search strategy may have overlooked some relevant studies. For example, the Kapsetaki and Easmon [4] review also found and excluded studies examining ED in visual artists, a population not captured in this review. This reflects broader challenges in the field, where research on non-performance artists remains fragmented and sparse.

Fourth, inter-rater reliability during the screening stages was extremely low, including negative κ values, and this constitutes a serious methodological limitation of the review process. While these values may partly reflect well-known limitations of κ in contexts with highly imbalanced inclusion decisions or low prevalence of eligible studies, as well as heterogeneity and inconsistent reporting across the primary literature, this does not negate the concern regarding low agreement between reviewers. Accordingly, this limitation is acknowledged explicitly, and the findings should be interpreted with appropriate caution. Discrepancies were resolved through detailed discussions among reviewers and consensus decision-making; however, low initial agreement may still have implications for the robustness of the study selection process.

Notwithstanding these limitations, this is the first review to systematically synthesise evidence on EDs in non-dance artistic populations and to include a meta-analysis, addressing a critical gap in the literature. The use of systematic methods, predefined inclusion criteria, and quality appraisal enhances transparency and provides a more reliable foundation for future research.

### Clinical implications

The findings underscore the importance of recognising how artistic identity and occupational context shape the presentation and treatment of ED symptoms, body image concerns and related general psychopathology. Artists may present with heightened perfectionism, body dissatisfaction, and preoccupation with performance-related appearance standards, which can intensify core ED symptoms such as restrictive eating, binge–purge behaviours, and body image disturbance. Although our review has shown that anxiety and mood disorders frequently co-occur with EDs in this population, the nature and mechanisms of this comorbidity remain poorly understood and warrant further investigation. Assessment approaches that consider occupational stressors alongside personal strengths—such as creativity, emotional expressiveness, and social connectedness [6]—may support more nuanced case formulations. Integrating these protective factors into treatment could foster resilience and contribute to improved therapeutic outcomes.

Beyond the clinical setting, the findings reveal structural gaps in mental health provision for artists. The elevated prevalence of EDs calls for preventive and early intervention strategies embedded within arts education and professional environments. Integrating mental health education, body image literacy, and accessible ED screening into training programs could mitigate risk before it escalates. At the professional level, embedding affordable and stigma-free resources within arts organisations, unions, and funding bodies is crucial, particularly given the financial instability many artists face. Policies that prioritise occupationally relevant support—such as addressing performance anxiety, body-related evaluation, and employment precarity—can reduce barriers to care and promote sustainable careers in the arts.

## Conclusion

This review highlights consistent evidence that artists are at elevated risk for EDs, marked by higher rated of ED-related outcomes, greater ED symptom severity, body image concerns, and comorbid anxiety and depression. Although limited by methodological weaknesses, the findings suggest that artists may constitute a vulnerable population requiring tailored clinical and policy attention. Future research should use rigorous diagnostic assessments, standardised measures, and larger, more diverse samples to clarify ED occurrence and symptom patterns across artistic individuals. Strengthening the evidence base will not only enhance scientific understanding of EDs in artists but also inform more effective prevention, treatment, and resource allocation strategies that address the specific needs of this understudied population.

### What is already know on this subject?

Eating disorders (EDs) arise from interacting sociocultural, familial, and individual vulnerabilities, including thin-ideal pressures, perfectionism, low self-esteem, and maladaptive cognitions. Artists may be particularly exposed to these risks, as evidence indicates elevated levels of depression, anxiety, perfectionism, and self-criticism in creative populations, all of which are linked to eating pathology. However, research has largely focused on dancers, with limited and methodologically inconsistent evidence regarding ED prevalence and associated psychopathology across the broader artist population.

### What this study adds?

This study provides the first comprehensive synthesis of ED prevalence, risk, body image concerns, and associated psychopathology across a broad range of artistic populations beyond dance. By integrating findings across diverse creative fields, it demonstrates that artists may experience elevated ED risk and related vulnerabilities, while also highlighting substantial methodological heterogeneity and reliance on self-report assessments. These findings clarify the current evidence base and underscore the need for more rigorous, clinically validated, and longitudinal research to inform targeted prevention and intervention efforts.

## Supplementary Information

Below is the link to the electronic supplementary material.

Supplementary file 1.

Supplementary file 2.

## Data Availability

All data is included in the manuscript.
