# Supplementary material for: Artists and eating disorders: what do we know? A systematic review of the evidence and gaps
Source: Eat Weight Disord. 2026 May 5;31(1):64. doi: 10.1007/s40519-026-01863-3 (PMC13287114; doi:10.1007/s40519-026-01863-3)
Supplement: Supplementary file 1 [file 40519_2026_1863_MOESM1_ESM.docx]

**Supplementary Materials 1**

**Search terms**

The following is the full search terms used for searching the three databases.

PsycINFO and Medline:

1. eating disorders/ or anorexia nervosa/ or "avoidant/restrictive food intake disorder"/ or binge eating disorder/ or bulimia/ or feeding disorders/

2. body dissatisfaction/ or body image/

3. (eating disorder* or body dissatisfaction or unhealthy weight control or restrained eating or binge-eating disorder* or binge eating disorder* or unspecified feeding or eating disorder* or "feeding and eating disorder*" or "feeding or eating disorder*" or anorexia nervosa or bulimia nervosa).mp.

4. (((body or appearance or physical or weight) adj4 concern*) or body dysmorphi* or (body adj4 (satisf* or dissatisf* or perception* or appreciation*)) or ((body or bodies) adj4 (image or images)) or physical appearance).mp.

5. physical appearance/

6. body dissatisfaction/

7. 3 or 4

8. 1 or 2 or 5 or 6

9. 7 or 8

10. creative professionals/ or artists/ or musicians/ or writers/ or architects/

11. (musical* or musician* or instrumentalist* or performer* or singer* or orchestra* or vocal* or "voice or choir*" or creativ* or artist* or opera or theatre or actor* or acting or drama or comedian* or magician* or mime or poet*).mp.

12. 10 or 11

13. 9 and 12

14. binge eating/ or dietary restraint/

15. 9 or 14

16. 12 and 15

Web of Science:

TS=("eating disorder*" OR "body dissatisfaction" OR "unhealthy weight control" OR "restrained eating" OR "binge-eating disorder*" OR "binge eating disorder*" OR "unspecified feeding or eating disorder*" OR "feeding and eating disorder*" OR "feeding and eating disorder*" OR "anorexia nervosa" OR "bulimia nervosa")

OR

(((TS=("(body OR appearance OR physical OR weight) NEAR/3 concern*")) OR TS=("body dysmorphi*")) OR TS=("body NEAR/3 (satif* OR dissatif* OR perception* OR appreciation*)")) OR TS=("(bodies OR body) NEAR/3 (image OR images)"))

AND

TS=(musical* OR musician* OR instrumentalist* OR performer* OR singer* OR orchestra* OR vocal* OR voice OR choir* OR creativ* OR artist* OR opera OR theatre OR actor* OR acting OR drama OR comedian* OR magician* OR mime OR "creative professional*" OR writer* OR architect* OR poet*)
