# Supplementary material for: Artists and eating disorders: what do we know? A systematic review of the evidence and gaps
Source: Eat Weight Disord. 2026 May 5;31(1):64. doi: 10.1007/s40519-026-01863-3 (PMC13287114; doi:10.1007/s40519-026-01863-3)
Supplement: Supplementary file 2 [file 40519_2026_1863_MOESM2_ESM.docx]

**Supplementary Material 2: Exploratory Meta-Analyses**

The meta-analyses presented in this Supplementary Material are preliminary and should be interpreted with caution. They are intended to be exploratory and illustrative rather than confirmatory. Given the substantial heterogeneity across studies, differences in study designs, and variation in assessment tools, these analyses do not provide definitive or population-representative prevalence estimates.

Accordingly, pooled estimates—particularly the pooled estimate of eating disorder (ED) risk—should not be interpreted as reliable summary prevalence estimates or used for population-level inference. Instead, these analyses are included to provide an indicative synthesis of the literature, illustrate variability across studies, and highlight key methodological limitations and gaps to inform future research.

**Meta-Analysis**

The meta-analysis was conducted using the meta package in R 4.4.3. Publication bias was assessed using a graphical method. Additionally, sensitivity analysis was conducted to assess the influence of included studies. Pooled prevalence was presented along with a 95% Confidence interval (CI) using a forest plot and table. Furthermore, a meta-regression analysis was conducted to examine the moderating effect of age, sex, and profession categories. However, due to the limited number of studies, only a bi-variable regression was conducted, entering one variable at a time.

**Pooled prevalence of ED risk**

Figure 1 shows a forest plot of a meta-analysis to estimate the pooled prevalence of ED risk among artists, synthesising eight studies (DiPasquale, 2014; Joseph 1982; Kapsetaki 2019; Szabo, 2019; Uriegas, 2021; van Rens, 20222; Vitzthum, 2013; Woropay-Hordziejewicz 2022). Based on the analysis, the pooled prevalence of ED risk was found to be 13.8% (*n* = 8; 95% CI: 8.1-22.4*; I^2^* = 94.1%; *p* < 0.01). However, a high heterogeneity remained among studies.

## Table 1 presents the subgroup analysis output pooling prevalence of ED risk across mean age (mean age < 25 yr Vs mean age ≥ 25 yr), proportion of female participants (< 50% Vs ≥ 50%), and professional category of sample population (music Vs performance artists) groups. Based on the sub-group analysis studies with mean age ≥ 25 yr reported a higher prevalence of ED risk (*n = 3*; prevalence = 22.7%; 95% CI: 14.9-33.2; *I^2^ =* 96.5%) than studies with mean age < 25 yr (*n* = 5; prevalence = 9.4%; 95% CI: 4.8-1.8; *I^2^* = 81.3%). On the other hand, studies with ≥ 50% female participants reported higher ED risk prevalence (*n =* 4; prevalence = 18.6%; 95% CI: 11.5-28.7; *I^2^* = 96.3%) than studies with < 50% females (*n =* 3; prevalence = 7.3%; 95% CI: 2.4-20.1; *I^2^* = 91.7%).

Furthermore, performance-based artist studies reported a higher prevalence of ED risk (*n* = 4; prevalence = 19.8%; 95% CI: 11.6-31.7; *I^2^* = 94.6%) than music artist studies (*n* = 4; prevalence = 10.8%; 95% CI: 4.6-20.4; *I^2^* = 88.7%). However, none of the subgroup analyses reduced the heterogeneity level observed between the included studies. Moreover, sensitivity analysis revealed no influential study that affected the pooled prevalence as well as the heterogeneity level.

**Small study effect**

Visual inspection of funnel plots (see Figure 2) and trim-and-fill analyses was conducted; however, given the small number of included studies (n < 10), these results should be interpreted with considerable caution, as these methods have limited power in such contexts and may yield unstable or misleading estimates of small-study effects. Accordingly, these analyses are reported for completeness and should be considered exploratory. As shown in Figure 2, the funnel plot suggests slight asymmetry toward the negative side; however, formal statistical testing (e.g., Egger’s test) was not performed due to the small number of studies. A trim-and-fill analysis imputed three additional studies, yielding an adjusted pooled prevalence of 22.6% (n = 11; 95% CI: 12.0–38.3; I² = 94.6%; p < .01). These results should be interpreted cautiously and not taken as firm evidence of publication bias.

**Moderation analysis**

Table 2 presents a moderation analysis output including proposed moderators, their estimated effect size with 95% CI, and significance level. The meta-regression analysis was conducted to assess the moderation effect of mean age, female sex composition, and professional categories. Due to the small number of studies, we included only one factor in the regression model at a time. Since one study (Joseph 1982) did not specifically indicate the proportion of female participants, a meta-regression for sex was undertaken using the remaining 7 studies. The moderation analysis showed none of the factors moderated the observed difference in the prevalence of ED risk in our review.

**Table 1**

*Total and subgroup pooled prevalence of eating disorder risk among artists*

| **Description** | **Number of studies** | **Pooled prevalence** | **95% LCI** | **95% UCI** | **Heterogeneity *(I)^2^*** |
| --- | --- | --- | --- | --- | --- |
| **Total ED risk** | 8 | 13.8% | 8.1 | 22.4 | 94.1% |
| **Subgroup** |  |  |  |  |  |
| **Mean age** |  |  |  |  |  |
| **≥ 25 yr** | 3 | 22.7% | 14.9 | 33.2 | 96.5% |
| **< 25 yr** | 5 | 9.4% | 4.8 | 1.8 | 81.3% |
| **Female composition** |  |  |  |  |  |
| **≥ 50%** | 4 | 18.6% | 11.5 | 28.7 | 96.3% |
| **< 50%** | 3 | 7.3% | 2.4 | 20.1 | 91.7% |
| **Profession** |  |  |  |  |  |
| **Performance artists** | 4 | 19.8% | 11.6 | 31.7 | 94.6% |
| **Music artists** | 4 | 10.8% | 4.6 | 20.4 | 88.7% |

*Note.* ED= Eating Disorder

**Table 2**

*Meta regression of eating disorder risk prevalence*

|  |  | **estimate** | **se** | **tval** | **df** | **pval** | **CI.lb** | **CI.ub** |
| --- | --- | --- | --- | --- | --- | --- | --- | --- |
| **Yi ß Age** | | | | | | | | |
|  | Intercept | -1.2218 | 0.4051 | -3.0159 | 6 | 0.0235 | -2.2131 | -0.2305 |
|  | Mean age < 25 yr | -0.9583 | 0.5464 | -1.7538 | 6 | 0.1300 | -2.2953 | 0.3787 |
| **Yi ß Female composition** | | | | | | | | |
|  | Intercept | -2.4168 | 0.5477 | -4.4129 | 5 | 0.0069 | -3.8246 | -1.0090 |
|  | >= 50% | 0.9441 | 0.6866 | 1.3750 | 5 | 0.2275 | -0.8209 | 2.7091 |
| **Yi ß Art category** | | | | | | | | |
|  | Intercept | -2.1285 | 0.4215 | -5.0499 | 6 | 0.0023 | -3.1598 | -1.0971 |
|  | Performance | 0.7423 | 0.6059 | 1.2251 | 6 | 0.2664 | -0.7402 | 2.2248 |

**Figure 1**

*Forest plot of included studies and their pooled prevalence of eating disorder risk among artists*

**
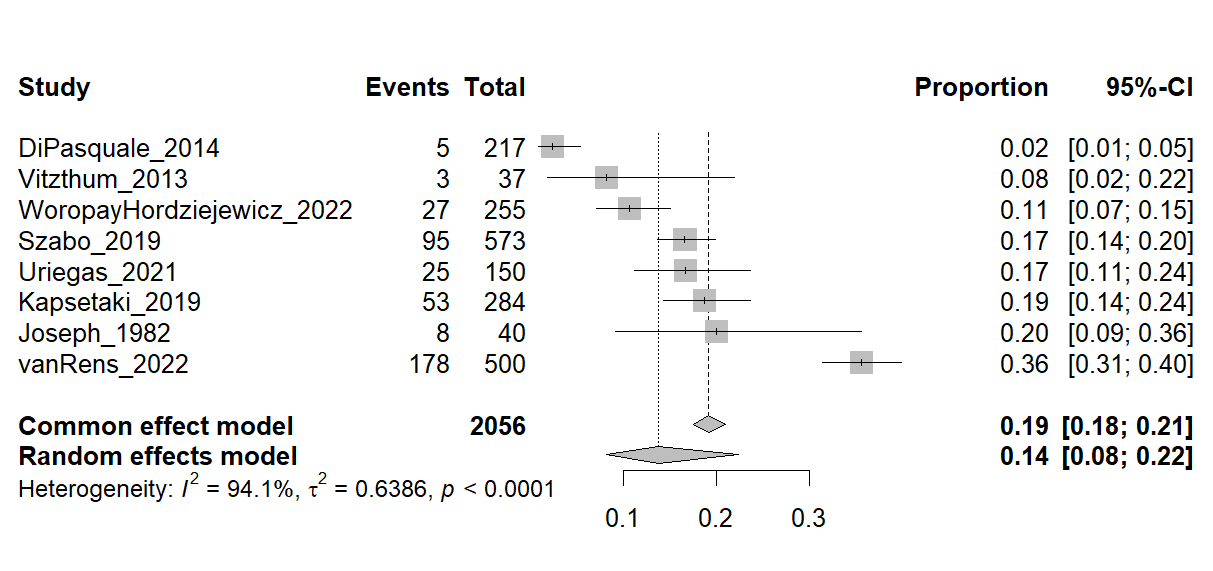
**

**Figure 2**

*Funnel plot of studies included in meta-analysis*

**
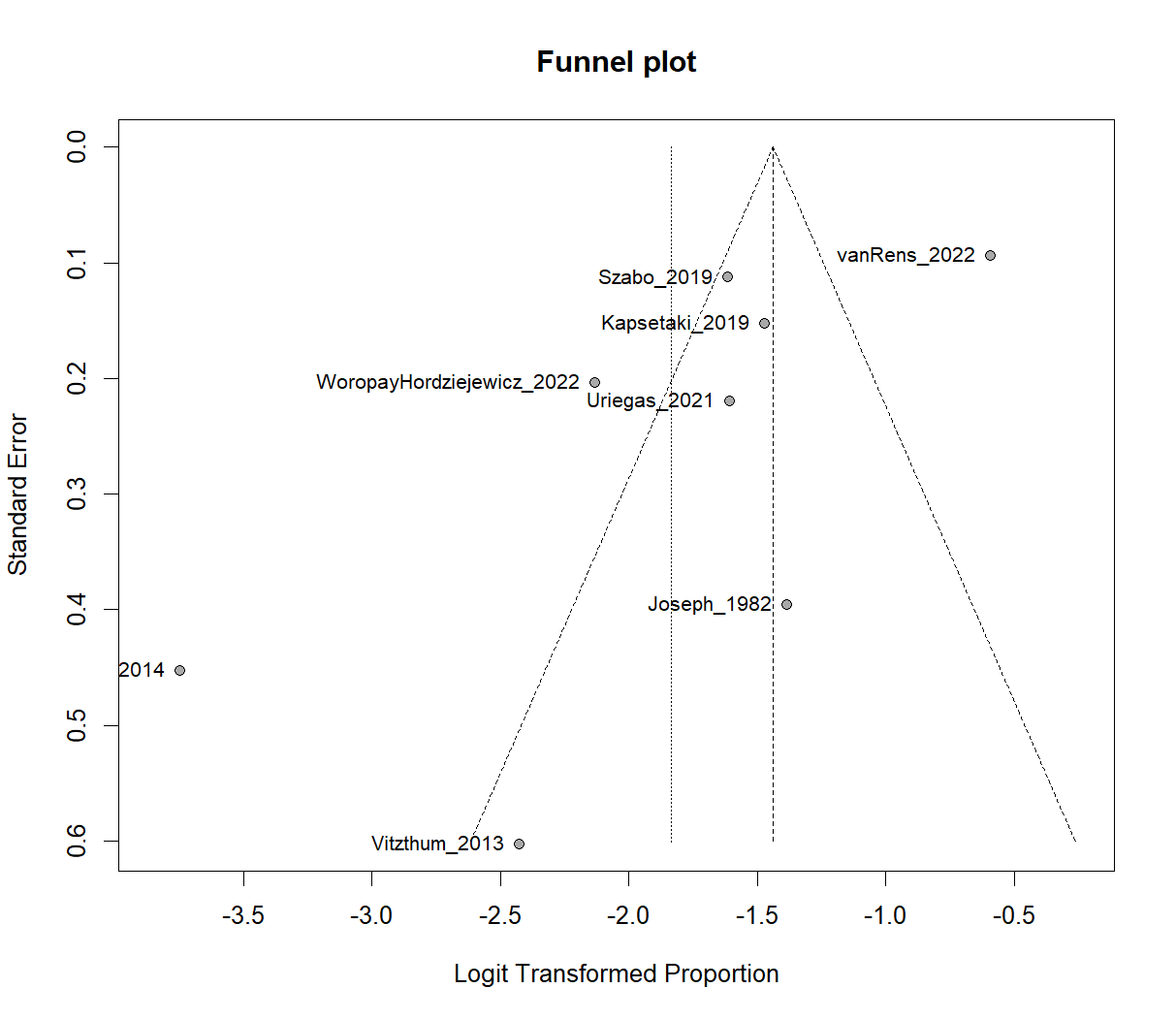
**
